# Bullous Pemphigoid and Other Pemphigoid Dermatoses

**DOI:** 10.3390/medicina57101061

**Published:** 2021-10-04

**Authors:** Valeryia Pratasava, Vikram N. Sahni, Aishwarya Suresh, Simo Huang, Abhirup Are, Sylvia Hsu, Kiran Motaparthi

**Affiliations:** 1 College of Medicine, Drexel University, Philadelphia, PA 19129, USA; valerie180896@gmail.com (V.P.); vns34@drexel.edu (V.N.S.); aisuresh97@gmail.com (A.S.); 2 Department of Dermatology, Lewis Katz School of Medicine, Temple University, Philadelphia, PA 19140, USA; simo.huang@tuhs.temple.edu (S.H.); sylvia.hsu@tuhs.temple.edu (S.H.); 3 College of Medicine, University of Florida, Gainesville, FL 32606, USA; acare2019@hotmail.com; 4 Department of Dermatology, College of Medicine, University of Florida, Gainesville, FL 32606, USA

**Keywords:** blistering, bullous, cicatricial, diagnosis, epidermolysis bullosa acquisita, mucous membrane, pemphigoid, vesiculobullous

## Abstract

The pemphigoid family of dermatoses is characterized by autoimmune subepidermal blistering. The classic paradigm for pemphigoid, and the most common member, is bullous pemphigoid. Its variable clinical presentation, with or without frank bullae, is linked by significant pruritus afflicting the elderly. Mucous membrane pemphigoid is an umbrella term for a group of subepidermal blistering dermatoses that favor the mucosal membranes and can scar. Epidermolysis bullosa acquisita is a chronic blistering disorder characterized by skin fragility, sensitivity to trauma, and its treatment-refractory nature. Clinicians that encounter these pemphigoid disorders may benefit from an overview of their clinical presentation, diagnostic work-up, and therapeutic management, with an emphasis on the most frequently encountered pemphigoid disease, bullous pemphigoid.

## 1. Introduction

Bullous pemphigoid (BP) is an autoimmune blistering disorder that predominantly affects the elderly. The median age for bullous pemphigoid is 80 years old, but it can also be observed in younger adults in drug-induced bullous pemphigoid and in the pediatric population [1]. In the United States the incidence of BP is between 2.4 and 23 cases per million in the general population each year, but in individuals over the age of 70, the number of cases annually is as high as 190–312 per million. The incidence rises exponentially with age with the highest burden of disease in people over age 80 [2]. The incidence of BP has been increasing in the past few decades due to the increased life expectancy of the aging population [3]. Women are affected more than men under the age of 75, but thereafter, the incidence is greater in men [2]. Multiple studies have shown that neurological conditions are risk factors as well as indicators of poor prognosis in bullous pemphigoid. Among others, some risk factors that have been identified throughout the literature include dementia, Parkinson’s disease, psychiatric disorders, and chronic bedridden conditions [2]. The mortality of patients with BP is compounded by the increased incidence of infectious complications which is the leading cause of death [4]. Although rare, pediatric BP has also been reported, and it presents differently than the adult form. Infants with BP have predominant acral involvement and almost always achieve full remission after treatment with systemic corticosteroids and single adjuvant therapy [4]. Lastly, pemphigoid gestationis (PG), also known as herpes gestationis or gestational pemphigoid, is a self-limited autoimmune bullous disease that classically occurs during late pregnancy and can cause transient blistering in the newborn. The antigenic target in PG mirrors that of BP, and it can be considered a variant of BP occurring in pregnancy. The differentiating factors mainly lie in the patient population affected and dermatosis onset during pregnancy [4]. 

Classified under the broader category of pemphigoid dermatoses are mucous membrane pemphigoid (MMP, also known as cicatricial pemphigoid) and epidermolysis bullosa acquisita (EBA). MMP is its own family of chronic autoimmune subepidermal blistering dermatoses differentiated from BP by its predilection for mucosal surfaces, potential for scarring, and different antigenic targets. MMP is categorized into classic, ocular, and anti-laminin 332 types [5]. EBA represents another acquired subepidermal blistering dermatosis characterized by autoantibodies against collagen VII and is noted for its chronic and treatment-refractory nature [6]. Overall, MMP and EBA are encountered much less frequently than BP, although exact incidence rates are unknown. A study of the incidence of subepidermal blistering dermatoses in France noted that BP made up the overwhelming majority of cases, with an estimated 406–436 new cases per year. In comparison, MMP and EBA were estimated to have an incidence rate of 70 and 11–17 new cases per year, respectively [7]. 

For BP, the most common disorder in the pemphigoid group, an understanding of the clinical presentation and pathophysiology are necessary for diagnostic work-up. Recent and upcoming therapies that target inflammatory mediators to improve the quality of life and reduce pruritus in elderly patients are reviewed. Clinicians who encounter less common pemphigoid disorders, such as MMP and EBA, also benefit from a review of stepwise therapeutic ladders and multidisciplinary approach to care.

## 2. Pathophysiology

### 2.1. Bullous Pemphigoid 

The primary pathophysiology of bullous pemphigoid involves the creation of autoantibodies to self-antigens in the basement membrane, specifically, BP230 and BP180 (also known as bullous pemphigoid antigen 1 and 2, respectively). BP230 is a cytoplasmic protein from the plakin family. It is a part of the hemidesmosome complex and is involved in anchoring intermediate filaments to the cytoskeleton [8]. BP180 is a transmembrane glycoprotein that is also a constituent of the hemidesmosome and spans the lamina lucida of the basement membrane zone. Its NC16A extracellular domain is regarded as the main antigenic epitope in bullous pemphigoid. IgG autoantibodies bind to BP180 and activate the inflammatory cascade [3]. These antigens play a central role in maintaining integrity of the hemidesmosome, thus securing the attachment of the epidermis to the dermis. Auto-antibodies against BP180 have been demonstrated to be responsible for dermal–epidermal separation and subsequent subepidermal blister formation in patients with BP. However, the pathogenicity of antibodies against BP230 is not as clear, and the titers of these antibodies do not correlate with disease activity [9]. Rather, BP230 antibodies likely develop as a result of epitope spreading. IgG1 autoantibodies are most prevalent in BP, but the titers of IgE antibodies are also often elevated in many patients [3]. Researchers have shown that injection of BP IgE into human skin grafts resulted in formation of urticarial plaques that are often seen in the non-bullous phase of the disease [8]. Numerous studies have evaluated the potential role of IgE in the pathogenesis of BP. The role of omalizumab in treating BP has supported an IgE-mediated pathway. Dimson et al. found that elevated total IgE levels were found in 70% of untreated patients with BP, and 86% had anti-BP180-NC16A antibodies [10]. Messingham et al. found their assay detected IgE autoantibodies in 77% of sera tested [11]. IgE causes degranulation of mast cells and basophils, contributing to tissue damage and inflammatory pathways. Anti-BP180-NC16A IgE class autoantibodies also induce eosinophil recruitment by binding to basal keratinocytes in mouse models of BP [12].

Bullous pemphigoid is significantly associated with major histocompatibility class II allele HLADQB1*03:01 which is involved in the presentation of antigens to CD4+ lymphocytes [4]. These CD4+ cells in turn release interleukin-17 (IL-17) which can be detected in the lesions of patients with early BP. IL-17 plays a role in upregulating the release of neutrophil elastase and matrix metalloprotease-9 responsible for dermal–epidermal separation [9]. Patients with BP have increased levels of IL-4, IL-13, peripheral and lesional eosinophils, circulating IgE, and Th2-cell related activity, signaling, and allergic type immune dysregulation when compared to healthy patients [11,13]. IgE and peripheral eosinophil levels may correlate with disease severity in patients with BP [11]. The role of Th2-cell type immune dysregulation is further supported by the success of dupilumab in treating patients with refractory BP. Through its ability to inhibit IL-4 and IL-13 release, dupilumab targets an important pathway by which BP symptoms are induced.

The pathophysiology of drug-induced bullous pemphigoid is attributed to certain medications that can act as haptens and induce an antibody response. Haptens bind to proteins in the lamina lucida and change their antigenic properties, thus stimulating an antibody response. The most common medications involved in drug-induced bullous pemphigoid are compounds with sulfhydryl groups (penicillamine, captopril, penicillins, furosemide, and certain cephalosporins), compounds with a phenol ring (certain cephalosporins and acetylsalicylic acid), angiotensin-converting enzyme inhibitors other than captopril, most non-steroidal anti-inflammatory drugs, immunomodulators such as vaccines, dipeptidyl peptidase-IV inhibitors, and TNF-α inhibitors [9]. Other triggers for bullous pemphigoid include ultraviolet radiation, trauma, and burns. 

Bullous pemphigoid may arise several months after immune checkpoint inhibitor therapy. Compared to classic BP, checkpoint inhibitor-induced BP presents more often with nonbullous lesions, acral involvement, and a prolonged pruritic phase. Anti-programmed cell death protein-1 (PD-1) inhibitors such as pembrolizumab, nivolumab, and cemiplimab or anti-programmed death-ligand 1 (PD-L1) inhibitors such as atezolizumab, avelumab, and durvalumab have been implicated in checkpoint inhibitor-induced BP. The mechanism of checkpoint inhibitor-induced BP is immune stimulation leading to increased activity of T- and B-cells against basement membrane antigens. Development of BP after therapy with PD-1/PD-L1 inhibitors has been associated with an improved response to tumor treatment [14]. Histopathologic findings are indistinguishable from classic BP. However, necrotic keratinocytes and a lichenoid interface tissue reaction may be observed [14].

Bullous pemphigoid is associated with other autoimmune disorders such as rheumatoid arthritis, dermatomyositis, Hashimoto thyroiditis, and systemic lupus erythematosus (SLE) [15]. The association of BP with certain neurological conditions such as multiple sclerosis, Parkinson’s disease, and Alzheimer’s disease has been attributed to the fact that bullous pemphigoid antigens (BP230 and BP180) are expressed in both skin and central nervous system (CNS) [3]. BP180 is expressed in the hippocampus, cortex, amygdala, and cerebellum while the dystonin gene codes for both BP230 (skin isoform) and the neuronal isoform, termed BPAG-1a [4]. Autoantibodies formed in CNS due to any kind of insult may cross-react with BP antigens found in the skin precipitating bullous pemphigoid [3]. Some studies have shown that BP has occurred 5–12 times more commonly in patients following the diagnosis of multiple sclerosis [3].

The pathogenesis of BP and PG share similarities with the development of antibodies against BP180 and BP230. An increased susceptibility for PG is associated with HLA-DR3 and -DR4 [16]. The development of an autoimmune response against placental basement membrane zone proteins with subsequent cross-reactivity to self-antigens in skin serves as the hypothesized driving force behind PG [16].

### 2.2. Mucous Membrane Pemphigoid and Epidermolysis Bullosa Acquisita

Bullous lesions in MMP result from antibodies directed against the basement membrane zone, much like BP. Generally, MMP has been classified into three types based on the antigenic target involved: 1. classic MMP—antibodies target the C-terminus domain of BP180; 2. ocular MMP—antibodies target the β_4_ subunit of the α_6_β_4_ integrin involved in the hemidesmosome complex; 3. anti-laminin 332 MMP—antibodies target laminin 332, a glycoprotein involved in basement membrane zone cohesion [5].

The pathogenesis of EBA involves the development of antibodies against collage VII, an important component of the anchoring fibrils that mediates attachment of the basement membrane zone to the papillary dermis. Susceptibility to development of these antibodies has been associated with certain HLA subtypes and other autoimmune diseases, notably inflammatory bowel disease, systemic lupus erythematosus, rheumatoid arthritis, and myeloma [17].

## 3. Clinical Features

### 3.1. Non-Bullous Pemphigoid

The non-bullous phase of bullous pemphigoid can easily be confused with other dermatologic conditions such as atopic dermatitis, irritant contact dermatitis, dermatitis herpetiformis, and urticaria. The lesions at this phase are intensely pruritic and appear in the form of eczematous patches and urticarial-like wheals. Vegetative plaques may develop in the intertriginous zones [15]. The lesions most commonly occur on the abdomen, thighs, axillae, and inguinal folds [3]. BP rarely demonstrates significant palmoplantar involvement; the contrary is seen in the pediatric population [18]. The non-bullous phase of BP may last for months to years before progression to the bullous phase. Studies have reported that urticarial bullous pemphigoid develops into bullae quicker than eczematous form, or in about 6 weeks [4]. In skin of color, erythema is harder to discern while dyspigmentation is more common [19].

One rare manifestation of bullous pemphigoid is exfoliative erythroderma. This manifestation is easily confused with erythroderma caused by other diseases like atopic dermatitis and psoriasis. Patients present with generalized skin desquamation and skin erythema [3]. Other atypical variants of bullous pemphigoid include localized lesions like that seen in pretibial pemphigoid or have an uncommon morphology such as nodules seen in pemphigoid nodularis [15]. Prospective cohort studies have shown that more than 80% of patients are diagnosed with bullous pemphigoid at the bullous phase where vesiculobullous lesions are readily visible. Thus, there is a significant minority of patients that present with non-bullous pemphigoid, necessitating an awareness of this presentation in order to establish early diagnosis and initiate timely treatment [18]. In patients with non-bullous disease, the autoantibody titers are usually low, not sufficient enough to induce bullae formation. 

### 3.2. Classic Bullous Pemphigoid

**Figure 1:**
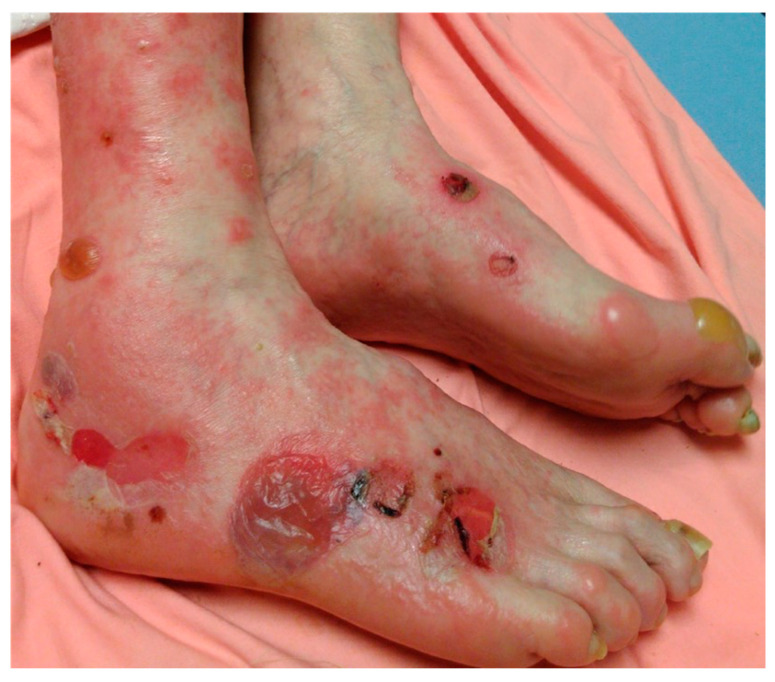
Bullous pemphigoid. Tense bullae overlying erythematous to urticarial plaques on acral skin.

In the bullous phase of the disease, tense vesiculobullous lesions appear most commonly on an erythematous or urticarial base. They may appear a few weeks to months after the non-bullous phase [4]. The bullae are tense (Figure 1) and do not break easily due to their subepidermal location. They usually appear symmetrically and predominate on flexural areas. The lesions can be annular, erythematous, urticarial, or eczematous with crusts and erosions. Oral mucosal involvement is seen in 10–30% of affected patients [15]. After the bullae heal, post-inflammatory hyperpigmentation can occur without scarring. Milia are commonly observed at sites of prior bullae or erosions. Vesicles and bullae may be serous or hemorrhagic and can appear in clusters or become widespread [20]. Herpetiform lesions can appear and present in an annular configuration [9]. Blisters can also arise in co-existence with lichen planus, and this presentation is termed lichen planus pemphigoides. Histopathologically, these lesions demonstrate features of both lichen planus and BP. The pathophysiology of this BP variant is thought to be due to unmasking of self-antigens as a result of damage to the basement membrane secondary to lichenoid inflammation. In BP of infancy, up to 84% of children present with moderate to severe bullous pemphigoid and all have acral involvement [21]. Additionally, acral involvement is much more common in infancy compared to childhood, 79% versus 17%, respectively [22]. In the pediatric population, vesicles develop in the genital area in 40% of cases, while in adults this number is closer to 9%. Knowledge of the pediatric presentation of BP can help avoid confusion with herpes simplex and sexual abuse [20]. Rarely, erosions and blisters of BP can be localized to one specific area of the body without widespread involvement of other body parts. This variant of BP can be triggered by certain agents (radiation, surgical procedure, transplant), but often the cause is unknown. PG, a variant of BP occurring in pregnancy, shares a similar clinical presentation to BP. Both non-bullous and bullous morphologies are seen, with rash onset usually occurring after week 20 of pregnancy [16].

### 3.3. Mucous Membrane Pemphigoid and Epidermolysis Bullosa Acquisita

Individual lesions of MMP and EBA cannot be reliably differentiated from BP. Vesiculobullous lesions are seen in all cases, but the distribution and ancillary findings can aid in distinction. In contrast to BP, MMP has oral involvement in up to 85% of cases. Mucous membranes are involved as the rule, not the exception in MMP, with the oral mucosa as the most common site and ocular conjunctiva as the second most common. The head and upper body are the most commonly involved cutaneous surfaces, and clinical evidence of scarring can lend credence to the diagnosis [5].

EBA presents clinically as the classical mechanobullous type or the nonclassical BP-like type [23]. In the mechanobullous type, vesiculobullous lesions are seen often on extensor surfaces and trauma-prone sites with associated scarring, milia, and skin fragility. BP-like EBA is indistinguishable from BP but can have an atypical distribution on the face. In a meta-analysis, 23% of patients with EBA had mucosal involvement, most commonly the oropharynx [24]. In contrast to BP, patients with EBA are diagnosed at a median age of 50 years [24]. EBA is usually chronic and finding an effective treatment can be challenging. Due to its low prevalence and the lack of randomized clinical trials, there are no consensus guidelines on treatment. 

A summary of clinical and histopathologic findings of pemphigoid dermatoses along with treatment options are summarized in Table 1.

## 4. Diagnosis

**Figure 2:**
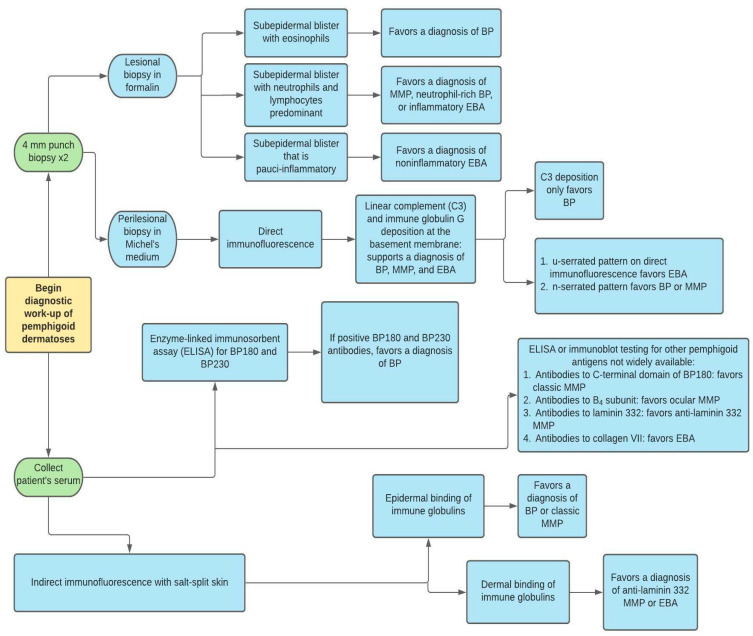
Diagnostic work-up of pemphigoid dermatoses. BP = bullous pemphigoid, MMP = mucous membrane pemphigoid, EBA = epidermolysis bullosa acquisita.

Diagnosis of pemphigoid dermatoses is made by clinical correlation with histopathologic, immunopathologic, and serologic features. In the evaluation of suspected BP, MMP, and EBA, two 4 mm punch biopsies should be taken, as shown in Figure 2. One biopsy is from the lesion itself and is used for routine staining and processing with hematoxylin and eosin (H&E). The other biopsy is taken from perilesional, intact skin near the blister and is sent for direct immunofluorescence (DIF) using Michel’s medium [25]. Although clinicians tend to follow traditional guidelines which point to perilesional biopsy for DIF, recent studies have questioned whether this practice is truly the most effective for BP. In a large retrospective chart review of 260 DIF biopsies in patients with suspected BP, clinicians obtained a positive DIF result from lesional non-bullous skin more often than from perilesional or normal skin (*p* = 0.004) [26]. 

**Figure 3:**
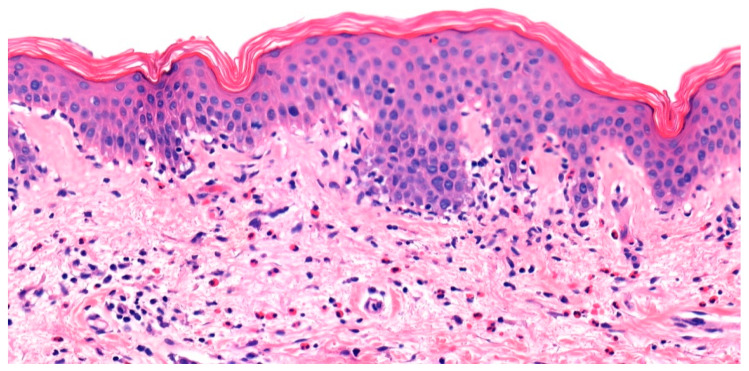
Urticarial bullous pemphigoid. Eosinophilic spongiosis characterized by pseudovacuolar change, eosinophil exocytosis, and dermal eosinophilia.

Histopathologic and immunopathologic findings play a crucial role in the diagnosis of BP. Although subepidermal clefting, separation, or splitting is classically observed, dermatopathologists should avoid the pitfall of solely relying on this feature for diagnosis. Hodge et al. found that in an examination of 81 slides sent for DIF of suspected BP, subepidermal blistering was observed in only 54.3% of cases. Focal intraepithelial clefting, re-epithelialization, non-bullous urticarial or eczematous pemphigoid, and blister roof necrosis were other observed pathologic features [27]. This histopathologic variability can be attributed to the spectrum of presentation of BP ranging from early, non-bullous phase to necrotic blistering with dyskeratosis. Common histopathologic features include the presence of a primarily eosinophilic infiltrate in the dermis and/or eosinophilic spongiosis (Figure 3) [3]. Although these features are nonspecific and can be seen in a variety of dermatologic conditions including arthropod bites, drug eruptions, or allergic contact dermatitis, correlating these findings with the clinical presentation and history can aid in the diagnosis of BP. Rarely, a neutrophil-rich infiltrate can be seen on histopathology in BP. MMP lesions will show a subepidermal blister with a predominantly neutrophilic and lymphocytic infiltrate. Eosinophils are variable, from a few to numerous per high-power field. Characteristically, lamellar fibrosis can be seen underneath the subepidermal bullae and can help distinguish MMP from other subepidermal blistering dermatoses, but this finding is not always present [28]. Biopsy of EBA shows two histopathologic patterns: 1. classic noninflammatory type; 2. inflammatory type with mixed neutrophils, lymphocytes, and variable eosinophils. A subepidermal blister is seen in both forms, but further testing is needed to distinguish EBA from other subepidermal blistering dermatoses [29].

Direct immunofluorescence (DIF) is considered the gold standard for evaluation of many blistering dermatoses. In BP, MMP, and EBA, the DIF pattern demonstrates linear deposition of IgG and complement proteins (C3) at the basement membrane zone (occasionally IgM or IgA have been reported). Unfortunately, DIF is not able to reliably distinguish the pemphigoid dermatoses [29]. C3 deposition without IgG can be seen in early disease of BP. DIF serration pattern can also help in differentiation of BP from EBA. The u-serrated pattern is found in collagen VII-targeting dermatoses including EBA and bullous systemic lupus erythematosus. A retrospective and prospective analysis of 291 biopsies of patients found that every case of EBA confirmed by serology had a u-serrated pattern [28]. 

Quantification of serum anti-BP180 and anti-BP230 antibodies by enzyme-linked immunosorbent assay (ELISA), is also routinely used for BP. Due to its high positive predictive value and low negative predictive value in BP specifically, ELISA should not be used as the sole and primary test in diagnosis [30]. Serologic testing for MMP and EBA antigens is not widely available, but some commercial laboratories do offer additional tests such as ELISA for collagen VII, the C-terminal domain of BP180, and laminin 332 [31]. 

Indirect immunofluorescence using salt-split human skin can also be useful in diagnosis. The salt-split technique can aid in differentiation between BP and other subepidermal blistering dermatoses like anti-laminin 332 MMP, EBA, and anti p-200/laminin γ1 pemphigoid. Antibodies in BP, classic MMP, and ocular MMP display an epidermal (“roof”) binding pattern. Patients with anti-laminin 332 MMP, EBA, and anti p-200/laminin γ1 pemphigoid exhibit antibody deposition in a dermal (“floor”) binding pattern [32].

It is necessary to use multiple modalities of testing in order to maximize sensitivity of objective measurements. In a retrospective study of 313 patients with BP compared to 488 controls, it was found that DIF exhibited the highest sensitivity in diagnosis (90.8%). Sensitivities for ELISA BP180 and BP230 autoantibody quantification were 72% and 59%, respectively. IIF performed using rabbit esophagus was found to be more sensitive than monkey and salt-split skin (76%, 73.2%, 73.3%, respectively) [33]. It is important to note that other studies have found ELISA sensitivity to be in the 80–90% range for BP [34,35].

Given the potential for widespread involvement and significant morbidity, it is necessary to characterize and evaluate the severity of BP using validated tools such as the Bullous Pemphigoid Disease Area Index (BPDAI). This scoring system quantifies the number and size of mucosal lesions, erythematous non-bullous lesions, and bullous lesions. Furthermore, the BPDAI contains a component for pruritic intensity [36]. When compared head-to-head with other scoring systems such as the Autoimmune Bullous Skin Disorder Intensity Score (ABSIS), BDPAI correlated better with physician global assessment (PGA), pruritic scales, and Autoimmune Bullous Disease Quality of Life (ABQOL) intraclass reliability [37].

The differential diagnosis for BP is extensive. Due to the various stages in which BP can present, it often goes undiagnosed or misdiagnosed until a severe blistering outbreak occurs. In the early, non-bullous stage it can mimic other conditions such as allergic contact dermatitis, atopic dermatitis, prurigo nodularis, drug reactions, urticaria, and urticarial vasculitis. In the bullous stage, BP can be misdiagnosed as other subepidermal blistering dermatoses like linear IgA bullous dermatosis (LABD), dermatitis herpetiformis, EBA, MMP, anti p-200/laminin γ1 pemphigoid, bullous lupus erythematous, and fixed drug eruption. The histopathologic and laboratory findings in PG mirror that of BP. Ultimately, the clinical context of a pregnancy differentiates PG from BP. IIF with salt-split skin and ELISA can aid in the differentiation of BP from MMP and EBA. LABD can show similar H&E findings to neutrophil-rich BP. DIF remains the gold standard for LABD diagnosis as linear IgA deposition at the basement membrane is observed in all cases [38]. Anti-p200/ laminin γ1 pemphigoid is a rare, scantily characterized blistering condition that was once thought to be a variant of BP. Similar DIF findings of linear deposition of IgG and C3, n-serration, and mixed reliability of IIF in anti-p200 pemphigoid often result in misdiagnosis. Immunoblot analysis can help isolate the target protein (laminin γ1) to confirm the diagnosis [2].

## 5. Management

BP lesions can last from months to years depending on severity. The treatment goal is symptom control with minimal side effects. Although some cases are self-limiting, lesions may take months to years to resolve and according to a retrospective study conducted in the UK, the mortality rate of patients with BP is double that of the control population of healthy adults [1]. Some studies have reported a 1-year mortality rate of 19% for patients with BP in the United States and in the United Kingdom [1,39]. First-line treatment for BP includes high potency topical corticosteroids [1]. Topical corticosteroids, such as 0.05% clobetasol propionate cream can be used twice daily on the entire body if lesions are widespread (sparing the face). Generalized involvement typically necessitates 20–30 grams of clobetasol propionate cream per day. With localized disease, topical therapy can be lesional. Patients should be monitored for atrophy, striae, and telangiectasias. Extensive involvement historically required systemic treatment with oral corticosteroids, although the increased risk for morbidity and mortality due to systemic corticosteroids in the elderly population must be considered. In a study comparing outcomes of patients treated with topical steroids versus oral steroids, more patients with both moderate and extensive BP treated with topical corticosteroids achieved control of disease by day 21, compared to patients treated with oral steroids [40]. However, high potency topical steroids are typically avoided on the face, and compliance may be a challenge, especially in the elderly population. In the case of more widespread or generalized disease topical treatments may not be feasible, depending on the patient. In all such instances, systemic steroids such as oral prednisone 0.5–1 mg/kg/day can be considered [41]. Once symptoms are controlled, the regimen can be adjusted to a tapered dose. 

In patients with localized disease who achieve suboptimal treatment results with steroid therapy or have contraindications or adverse effects related to steroid therapy including osteoporosis, steroid-induced myopathy, sepsis, and thromboembolic events, other second line therapies with varying levels of evidence have been described [41,42]. These include dual therapy with an anti-inflammatory antibiotic like doxycycline with nicotinamide, sulfonamides, or topical immunomodulators. A randomized prospective study comparing the efficacy of doxycycline to prednisolone as initial therapy for BP indicated that the doxycycline was non-inferior to prednisolone in achieving short-term blister control defined as three or fewer significant blisters at 6 weeks of treatment [43]. Moreover, the study also found that patients treated with doxycycline had fewer severe and fatal events, suggesting that tetracyclines might be a more appropriate choice for patients with localized disease, comorbidities, or contraindications to oral steroid therapy. 

Second line therapies that can be added as adjunctive therapies to corticosteroid regimens for generalized BP include immunosuppressive treatments including azathioprine, mycophenolate mofetil, methotrexate, cyclophosphamide, cyclosporine, chlorambucil, and leflunomide. A study comparing patients treated with oral corticosteroids with adjunctive mycophenolate mofetil to patients treated with oral corticosteroids and adjunctive azathioprine showed both treatment groups were similar in achieving disease remission but that azathioprine had a significantly higher risk of hepatotoxicity, indicating that mycophenolate mofetil might minimize hepatic adverse effects while offering efficacious therapy [44]. An important factor to consider when using an immunosuppressive adjunct therapy is its side effect profile. For instance, cyclophosphamide can be carcinogenic and cause infertility. Thus, cyclophosphamide therapy is only considered when patients have failed therapy with steroids and adjunctive mycophenolate mofetil or azathioprine, experienced significant side effects with other therapies, or have BP that is quickly progressing [42]. Alternative or adjunctive therapies for generalized BP refractory to first line treatments include immunomodulators or biologics such as intravenous immunoglobulin (IVIg), plasmapheresis, and rituximab. In a study of 15 patients with BP refractory to other therapies, it was found that all patients achieved disease remission while being treated with IVIg. Moreover, there was a statistically significant increase in measures such as quality of life and reduction in number of hospitalizations and length of hospital stay, among other parameters in the patients treated with IVIG monotherapy when compared to other treatments [45].

**Figure 4:**
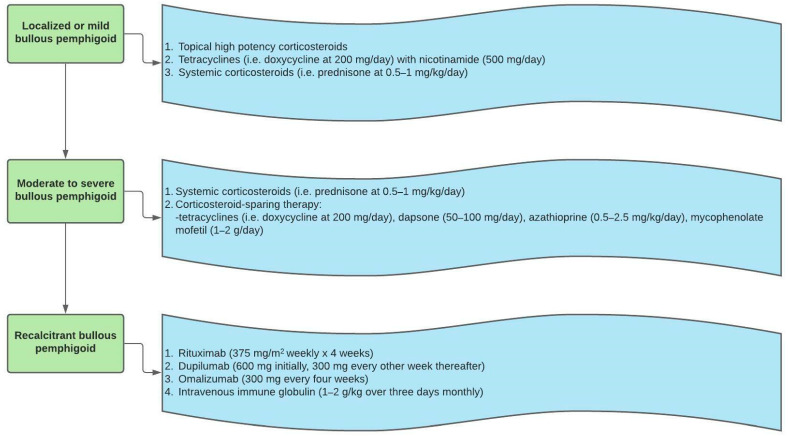
Bullous pemphigoid treatment algorithm.

More recent therapies for BP include rituximab, dupilumab, and omalizumab. Dupilumab targets the IL-4 receptor, rituximab targets CD20, and omalizumab targets IgE. One case series showed that out of 13 patients with refractory BP who received dupilumab, 12 achieved disease improvement, and 7 achieved symptom resolution [45]. Other case reports have also described successful use of dupilumab or combination therapy of dupilumab and omalizumab for recalcitrant BP [46,47,48]. These therapies have varying levels of evidence to support their use in patients with refractory BP. A major factor to consider is financial accessibility. The high cost of these novel therapies may limit access. The detailed algorithm for management of BP is presented in Figure 4. 

Another aspect of caring for patients with BP is proper skin and blister care. There is a paucity of data regarding blister care. During active disease, blisters should be left alone. For open or eroded areas, antiseptic baths, soaks, and bath oils can be helpful. Moreover, painful areas can be covered with low adhesion dressing so as not to irritate or harm the skin [49].

The treatment for PG centers on the balance between symptomatic management with minimal fetal risk. Mild disease is treated with mid-to-high potency topical corticosteroids. In more severe cases, systemic non-fluorinated corticosteroids are used at the minimal dose necessary to minimize exposure risk to the fetus. 

**Figure 5:**
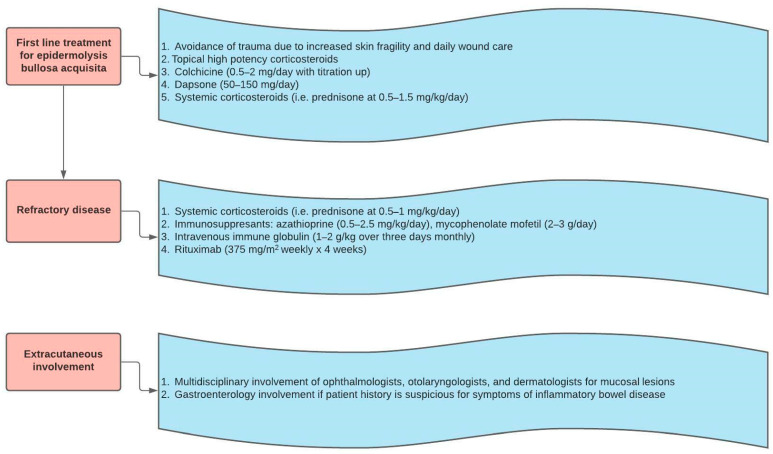
Epidermolysis bullosa acquisita treatment algorithm.

The therapeutic regimens for EBA and MMP have significant overlap with the treatments for BP. However, one of the important features that differentiates EBA and MMP from BP is the predilection for persistent extracutaneous involvement and subsequent need for multidisciplinary care. First line treatment for EBA emphasizes trauma avoidance as the skin is particularly fragile and prone to damage, especially on the hands and feet [50]. Given the rarity of the condition, there is a lack of high quality data regarding the comparative efficacy of treatments for EBA [50]. Other treatments that are considered to be first line for EBA include high potency topical corticosteroids, colchicine, dapsone, and systemic corticosteroids [6]. For disease that is recalcitrant to these therapies, immunosuppressants such as azathioprine and mycophenolate mofetil, IVIg, and rituximab are second line options. In a literature review by Engineer et al., six out of seven patients with refractory EBA in various studies treated with IVIg achieved symptom reduction described as decreased new blisters and healed old lesions. Moreover, all patients who achieved symptom improvement did not experience any side effects and were able to reduce their concomitant use of adjuvant therapies [50]. Oktem et al. reported an overall reduction of mucocutaneous involvement and oral severity scores following combination therapy with IVIg and rituximab in five patients with refractory EBA [51]. A stepwise algorithm for EBA treatment is shown in Figure 5. 

**Figure 6:**
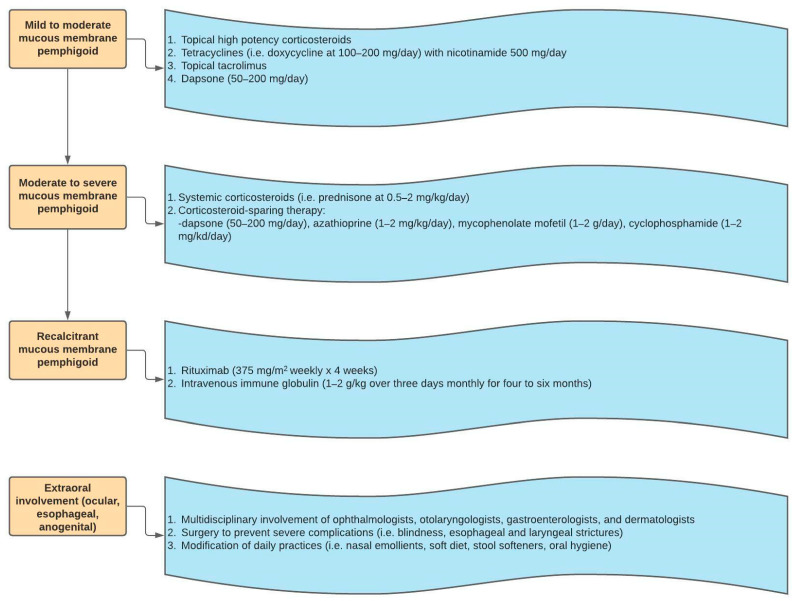
Mucous membrane pemphigoid treatment algorithm.

The treatments used for MMP are similar to those of BP and EBA. The therapy for MMP is selected based on the severity of symptoms and extent of involvement. If symptoms are mild to moderate, and limited to oral mucosal and/or skin involvement, then topical corticosteroids, tetracyclines and nicotinamide, topical tacrolimus, and dapsone can be utilized [5,52]. For more severe symptoms with ocular, laryngeal, nasopharyngeal or genital disease, systemic steroids or immunosuppressants such as azathioprine, mycophenolate mofetil, or cyclophosphamide can be considered [5,52]. Similar to recalcitrant EBA, in the case of refractory MMP, IVIg and rituximab can be utilized. Figure 6 shows the stepwise ladder for MMP management. For both MMP and EBA, multidisciplinary care is requisite when there is significant extracutaneous involvement. Significant sequelae from mucositis include conjunctival scarring, esophageal strictures, and laryngeal damage. Consequently, a multidisciplinary team composed of gastroenterologists, ophthalmologists, otolaryngologists, and wound care specialists may be needed to properly address the multisystem involvement of MMP and EBA [5]. 

## 6. Conclusion

For clinicians, the most commonly encountered dermatosis of the pemphigoid family is BP. Awareness of its varied clinical presentation and appropriate laboratory work-up facilitate fast and accurate diagnosis. While mortality has decreased with the advent of corticosteroid therapy, BP still carries significant morbidity and for many elderly patients, chronic corticosteroid use has significant risks as well. Newer immune-modulating therapies like dupilumab and rituximab can offer clinicians more options for steroid-sparing treatment. While MMP and EBA are significantly rarer diseases than BP, knowledge of their presentation and different treatment regimens can help clinicians deal with their challenging and multi-faceted issues as well.

## Figures and Tables

**Table 1:** Summary of clinical and histopathologic findings and treatment of pemphigoid dermatoses.

Pemphigoid Dermatoses	Clinical Findings	Histologic Findings	Treatment
Classic bullous pemphigoid	Pruritic nonbullous phase followed by tense vesiculobullous lesions on erythematous or urticarial base. Flexural involvement predominantly.	Subepidermal blister with primarily eosinophilic-rich infiltrate in the dermis and/or eosinophilic spongiosis.	Mild: topical high-potency steroids, tetracyclines. Moderate to severe: systemic corticosteroids, azathioprine, mycophenolate mofetil. Recalcitrant: rituximab, dupilumab, omalizumab, IVIg.
Mucous membrane pemphigoid	Vesiculobullous lesions and oral/ocular involvement in 85% of cases. Predominantly involves the head/neck and upper body.	Subepidermal blister with a predominantly neutrophilic and lymphocytic infiltrate and lamellar fibrosis.	Mild to moderate: topical high-potency steroids, tetracyclines, topical tacrolimus, dapsone. Moderate to severe: Systemic steroids, rituximab, IVIg.
Epidermolysis bullosa acquisita	Vesiculobullous lesions on extensor surfaces, often in areas of trauma, such as elbows and knees.	Two types: 1. Subepidermal blister that is pauci-inflammatory; 2. Subepidermal blister with eosinophilic-rich infiltrate, indistinguishable from bullous pemphigoid.	1st line: topical high-potency steroids, colchicine, dapsone. Refractory: systemic steroids, rituximab, azathioprine, mycophenolate mofetil, IVIg.
